# Effect of N-Methyl-D-Aspartate Receptor Antagonist Dextromethorphan on Opioid Analgesia in Pediatric Intensive Care Unit

**DOI:** 10.1155/2016/1658172

**Published:** 2016-10-27

**Authors:** Mohammed Naeem, Hala Al Alem, Ali Al Shehri, Majed Al-Jeraisy

**Affiliations:** ^1^Department of Pediatrics, Division PICU, King Abdulaziz Medical City, Riyadh, Saudi Arabia; ^2^King Saud Bin Abdulaziz University-Health Sciences (KSAU-HS), Riyadh, Saudi Arabia; ^3^King Abdullah International Medical Research Center (KAIMRC), Riyadh, Saudi Arabia; ^4^Department of Pharmacy, King Abdulaziz Medical City, Riyadh, Saudi Arabia

## Abstract

*Objective*. Pain control is an essential goal in the management of critical children. Narcotics are the mainstay for pain control. Patients frequently need escalating doses of narcotics. In such cases an adjunctive therapy may be beneficial. Dextromethorphan (DM) is NMDA receptor antagonist and may prevent tolerance to narcotics; however, its definitive role is still unclear. We sought whether dextromethorphan addition could decrease the requirements of fentanyl to control pain in critical children.* Design*. Double-blind, randomized control trial (RCT).* Setting*. Pediatric multidisciplinary ICU in tertiary care center.* Patients*. Thirty-six pediatric patients 2–14 years of age in a multidisciplinary PICU requiring analgesia were randomized into dextromethorphan and placebo. The subjects in both groups showed similarity in most of the characteristics.* Interventions*. Subjects while receiving fentanyl for pain control received dextromethorphan or placebo through nasogastric/orogastric tubes for 96 hours. Pain was assessed using FLACC and faces scales.* Measurements and Main Results*. This study found no statistical significant difference in fentanyl requirements between subjects receiving dextromethorphan and those receiving placebo (*p* = 0.127).* Conclusions*. Dextromethorphan has no effect on opioid requirement for control of acute pain in children admitted with acute critical care illness in PICU. The registration number for this trial is NCT01553435.

## 1. Introduction

Narcotics are the mainstay analgesics to control pain in the intensive care settings. However, patients frequently require escalating doses of narcotics for pain control leading to higher risks of side effects and complications [1]. Previously, literature has mentioned various adjunctive therapies including N-methyl-D-aspartate (NMDA) receptor antagonist dextromethorphan [2]. NMDA receptors are implicated in mediating a decrease in narcotic effect and hence, being an NMDA antagonist, dextromethorphan may supplement the pain control by narcotics but this has never been studied in a randomized controlled trial in children admitted in PICU and is still unclear [3, 4], although there had been reported results in adult population [5–8]. The aim of this prospective double-blind randomized controlled trial is to evaluate the role of dextromethorphan as an adjunct to narcotic analgesia in pediatric intensive care unit (PICU).

## 2. Methods

### 2.1. Conduct of Study

This interventional double-blind randomized clinical trial (RCT) was conducted between January 2011 and January 2015 at King Abdulaziz Medical City (KAMC), Riyadh, KSA, a 1500-bed medical university hospital. The PICU is a multidisciplinary unit admitting about 900 patients per year under 14 years of age. It was conducted observing the principles of Helsinki declaration. This study was approved by the Institutional Review Board and King Abdullah International Medical and Research Center (KAIMRC) and involved pediatric intensive care unit patients (2–14 years). This study is registered with clinicaltrials.gov (identifier: NCT01553435) and was approved by Saudi Food and Drug Administration to study off-label use of study medicine. The project was periodically audited and monitored by Clinical Trials Monitoring Unit of KAIMRC to ensure maintenance of standards according to Helsinki declaration and to ensure adherence to the approved protocol.

### 2.2. Primary Outcome and Definitions

The primary outcome in this study was the cumulative dose of fentanyl in subjects with intervention of either dextromethorphan or placebo.

*Hemodynamic instability* was defined as a clinical state requiring pharmacologic support to maintain mean blood pressure between 10th and 90th percentile according to age and and/or to maintain capillary refill at less than 2 seconds.

*Liver failure or dysfunction* was defined as an increase in serum levels of aspartate transaminase (AST) or alanine transaminase (ALT) by at least twice the normal reference results for age.

*Multiple organ failure* was defined as the presence of deranged organ function involving two or more organ systems.

*Total infusion dose* was defined as the amount of fentanyl received during 96 hours per kilogram of body weight.

*Total cumulative dose of fentanyl* was defined as total infusion dose plus total intermittent (prn) doses of fentanyl per kilogram of body weight during 96 hours.

*Adequate pain control* was defined as FLACC score of zero (comfortable and relaxed) and/or faces score of zero (no pain).* Tolerance* was defined as the state of adaptation in which fentanyl exposure led to diminution of analgesic effects over time [9].

### 2.3. Study Protocol, Randomization, and Concealment

Upon admission all the patients were assessed for eligibility to be enrolled in the study as per inclusion and exclusion criteria. Inclusion criteria were as follows: (1) patients admitted in PICU within 72 hours of admission and (2) patients receiving intravenous infusion of fentanyl. The exclusion criteria were as follows: (1) patients who are less than 2 years of age, (2) patients who are hemodynamically instable, (3) enteral route which was contraindicated for medication administration, and (4) patients with hepatic or renal failure/dysfunction or multiorgan failure. Verbal and written information was provided to eligible patients/parent(s)/guardian(s). Informed consent was obtained once they decided to participate. Subjects' biodata and consent were entered in hospital electronic order system for randomization. Sample size was calculated on the basis of an absolute enhanced effect of 75–100% and assuming 10% drop out rate our estimated sample size was total number of 36 to give study a power of 80%. 36 randomization numbers were allocated to two groups by random chance and each group received 18.

Subjects, parent(s)/guardian(s)/caregiver, investigators, pharmacists, nurses, and paramedics were kept blinded. The intervention in this study was administration of dextromethorphan via nasogastric or orogastric tube. The unblinded research pharmacist from KAIMRC prepared placebo medicine that was identical to dextromethorphan in physical properties.

### 2.4. Postenrollment Management

Intervention study medicine was administered every 8 hours through nasogastric or orogastric tube in the standard recommended doses of 0.3 mg per kilogram per dose by bedside nurse.

The total duration of intervention was 96 hours. During this time frame, we had criteria to remove subjects from study if one or more of the following circumstances arise: (a) if the narcotic infusion was discontinued, (b) if subject developed hemodynamic instability, (c) withdrawal request by parent(s)/guardian(s), and (d) if subject's Glasgow Coma Scale (GCS) fell below 5 or subject was declared dead.

Enrolled subjects were continuously monitored with PICU monitoring system and pain was assessed via FLACC (face, legs, activity, cry, and consolability) and/or faces scales by the staff who were well trained and experienced with the universally standard goal of achieving pain control with minimum dose of fentanyl [10–12]. Clinicians titrated the fentanyl infusion dose and administered intermittent bolus (prn) doses to maintain adequate pain control. Each subject's medical record was evaluated to record variables including age, weight, height, body mass index (BMI), gender, length of stay in PICU and hospital, PRISM score, admitting diagnosis, provision of antibiotics during study period, starting and end dose of narcotics, and biochemical profile. All the subjects received midazolam infusion as sedative. Moreover, subjects did not receive any other analgesic including acetaminophen, ketamine, and ketorolac, sedative, or paralytic agent.

### 2.5. Literature Search and Analysis

We searched literature in PubMed with mesh headings of analgesia, acute care, narcotics, fentanyl, morphine, adjunctive therapies, NMDA antagonists, and dextromethorphan.

We recorded the data on Microsoft Excel, version 2007. The data were then exported to IBM Statistical Package for Social Sciences Statistics, version 20 (IBM Inc., Armonk, New York, USA), for further analysis by intention to treat. Demographic variables were assessed using Kolmogorov-Smirnov test for distribution and reported as median with interquartile ranges (IQR) and groups were compared using Mann–Whitney test. Biochemical data and fentanyl doses group comparison are presented as means with standard deviation (SD) and groups were compared using Student's* t*-test. All the statistical tests were two-tailed. The significant level for results was set at *p* < 0.05. In subgroup analysis we utilized Bonferroni correction to extract *p* value.

## 3. Results

**Figure 1:**
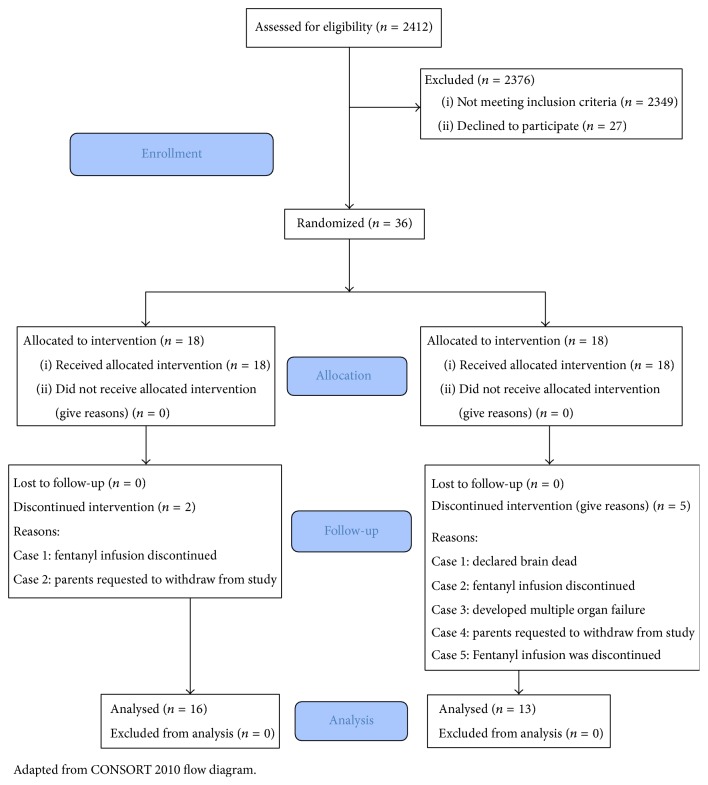
Flow diagram.

Figure 1 outlines the flow diagram. We assessed all the PICU admissions in our 20-bed multidisciplinary PICU from 2011 to 2015 for enrollment. 2412 patients were assessed for eligibility according to inclusion and exclusion criteria. Among these, 2376 patients did not meet the inclusion criteria. Additional 27 patients were not included in the study because parents/guardians declined to participate in the study. 36 subjects were enrolled via double-blinded randomization and at the end 18 were randomized to each of the two groups. Later on two patients were excluded from group 1. The reason in the first case was that the subject's fentanyl infusion was discontinued by clinicians and in the second case the parents requested withdrawal from study for unspecified reasons. Five subjects were removed before completion of intervention from group 2 with the following reasons: (1) subject was declared brain dead, (2) fentanyl infusion was discontinued, (3) subject evolved multiple organ failure, (4) parents requested withdrawal from study due to uncertainty about pain control, and (5) fentanyl infusion was discontinued. Hence, 16 patients in group 1 and 13 patients in group 2 were included in the analysis.

In Tables 1 and 2, we present the characteristics of the subjects in group 1 (dextromethorphan) and group 2 (placebo). The mean age in group 1 is higher than that in placebo group (9.7 versus 5.54; *p*, 0.0015; CI 1.74–6.56). There is statistically no significant difference regarding length of stay in PICU or hospital, PRISM score, use of antibiotics, and mean fentanyl infusions at the beginning and end of study enrollment. Group 1 received three patients with hematology/oncology problems, while group 2 did not. On the contrary, group 2 had one burn patient while group 1 did not have any burn patient. Otherwise, there was no significant difference between the two groups regarding the number of patients who had diagnoses of trauma, postoperative care, and respiratory failure. We recorded fentanyl dose at the beginning and at the end for all the subjects during study period of 96 hours and the analysis did not reveal any significant difference between the two groups. Regarding biochemical profile, there is no statistical difference between the two groups.

**Figure 2:**
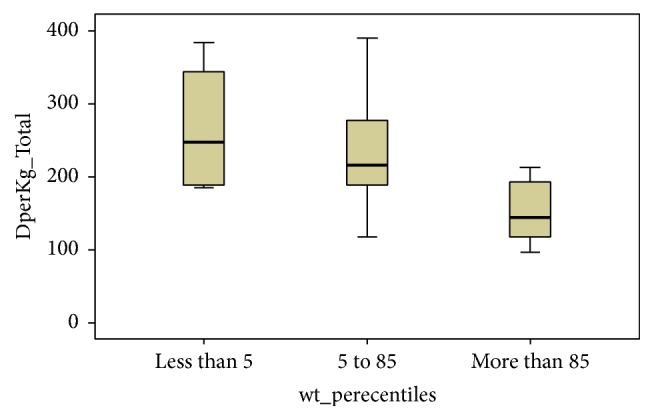
Variability of fentanyl dose requirements in subjects with different weight percentiles in dextromethorphan group.

In Table 3 the data presented are related to the comparison of fentanyl requirement in the two groups. We found no statistically significant difference in cumulative dose of fentanyl when administered with dextromethorphan (group 1) as compared to when administered with placebo (means, 217.9 versus 268; *p*, 0.127). Likewise, there is no statistical difference between the two groups regarding fentanyl whether administered as infusion or as prn bolus doses. This study found that when dextromethorphan was added, the accumulative requirements of fentanyl were less in subjects who were overweight (BMI more than 85%) as compared to the rest (Figure 2, *p* = 0.042).

## 4. Discussion

Multiple therapies had been mentioned in the literature which are considered adjunctive to narcotics. Some of these are beneficial to patients who have chronic pain such as antidepressants and anticonvulsant and some are beneficial to patients with acute pain such as acetaminophen and ibuprofen [13–20]. Regarding the role of dextromethorphan as adjunctive therapy in acute intensive care settings, our prospective double-blinded randomized interventional trial did not detect any significant effect of adding dextromethorphan on the required total accumulative dose of fentanyl for 96 hours of enrollment (means, 217.9 versus 268; *p*, 0.127).

Dextromethorphan (DM) is an antitussive drug. It is one of the active ingredients used to prevent coughs in many over-the-counter cold and cough medicines. DM is the D-isomer of the codeine analog of levorphanol but has little analgesic or addictive properties. However, it does act on the cough center in the medulla oblongata by elevating the threshold for coughing. In usual doses, the drug can cause dilation of pupils, but without significant reduction of respiratory rate. Dextromethorphan may cause slight elevations in blood pressure [21]. DM has also found other uses in medicine, ranging from pain relief to psychological applications. DM as a noncompetitive NMDA receptor antagonist can be used to block NMDA receptors in a dose-dependent manner, thus reducing the receptor binding sites available to opioids and to some extent producing an attenuation of opioid tolerance.

Dextromethorphan (DM) may play an important role in conditions of glutamate excitotoxicity (i.e., amyotrophic lateral sclerosis) because of its ability to suppress the overactivity of the glutamate system in the CNS [22]. In vitro studies have shown that both DM and its major metabolite (dextrorphan) noncompetitively antagonize N-methyl-D-aspartate (NMDA) and glutamate-induced excitation and excitotoxicity in the CNS and spinal regions. In addition, DM has been shown to inhibit NMDA-induced convulsions and attenuate hypoglycemic neuronal injury [23, 24].

The researchers have explored the role of dextromethorphan as adjunct to narcotics pain control in acute setting. Elliott et al. performed an animal study and showed that dextromethorphan decreased the tolerance to analgesic effect of morphine [3]. They showed that the requirement was much less on day 4 of morphine therapy when dextromethorphan was coadministered in comparison to placebo. Likewise, Price et al. studied oral doses of dextromethorphan (DM) in normal volunteer human subjects who rated intensities of first and second pain in response to repeated painful stimuli [25]. They demonstrated beneficial effect of dextromethorphan. Similarly, Mao et al. studied combined administration of dextromethorphan and morphine in rats and showed beneficial effect [4]. They concluded that combining DM with opiate analgesics may be a powerful approach for simultaneously preventing opiate tolerance and dependence and enhancing analgesia in humans. However, our study results are in contradiction to these previous studies. We strongly recommend a clinical trial with longer duration than 96 hours of intervention of dextromethorphan supplementary to narcotics for a definitive conclusion.

Our study has multiple limitations. Most notably it is single-centered, a possible effect on external validity. Despite efforts some cofounding factors might had been overlooked. Administration of dextromethorphan through nasogastric route and not intravenously may have an impact on our study results. In conclusion, this study did not detect any adjunctive effect of dextromethorphan when added to fentanyl for control of acute pain in children admitted with acute critical care illness in PICU.

In the future, we suggest a randomized controlled trial using higher doses of dextromethorphan preferably administered intravenously. We also suggest evaluating dextromethorphan's role in subset of particular patients having existing chronic pain due to underlying conditions such as patients with oncologic problems who get admitted in PICU for acute critical care illness.

## Figures and Tables

**Table 1:** Demographic characteristics. Clinical Outcomes of subjects in Groups 1 and 2 in RCT. Kolmogorov-Smirnov test used to assess distribution. nc, not computed.

Characteristics	Group 1: dextromethorphan (number = 16)	Group 2: placebo (number = 13)	*p* value
	Median (IQR)	Median (IQR)	
Age (Yr.)	9.7 (8–12)	5.54 (3.5–6)	0.0015
Gender: Male (number)	9	10	nc
LOS-PICU (days)	9 (6–21)	12 (6–24)	0.431
LOS-Hospital (days)	16.5 (9–46)	24.5 (12–61.5)	0.501
PRISM	17 (15–21)	18.1 (15.5–22)	0.75
Intubated (number)	16	13	nc
Underlying diagnosis (number)			
Trauma	10	10	0.57
Hem/onco	3	0	NA
Post-op	2	1	0.672
Respiratory failure	1	1	0.879
Burn	0	1	NA
Fentanyl infusion-start *µ*/kg/hr	2 (1–4)	2 (1–3)	0.16
Fentanyl infusion-end *µ*/kg/hr	3 (2–4)	3 (1–4)	0.7
Midazolam infusion-start mg/kg/hr	0.09	0.11	0.36
Midazolam infusion-end mg/kg/hr	0.16	0.18	0.27

**Table 2:** Biochemical variables of subjects in groups 1 and 2 in RCT. Groups compared via Student's *t*-test comparison of means. *p* < 0.05 is significant. Values are expressed as mean and standard deviation.

		Group 1: dextromethorphan (number: 16)		Group 2: placebo (number: 13)		*p *value^*∗*^
		Mean	±SD	Mean	±SD	
WBC (×10^3^/mm^3^)	Start	12.81	6.337	14.92	5.937	0.673
	End	11.13	3.879	11.31	3.225	0.894
Hemoglobin (g/L)	Start	103.56	16.252	112.23	12.544	0.104
	End	97.81	12.079	101.92	12.493	0.3747
Platelets (×10^3^/mm)	Start	302.31	169.591	362.54	166.289	0.348
	End	287.06	138.546	352.00	144.741	0.228
Aspartate transaminase (AST) (units/L)	Start	44.25	14.040	42.23	12.357	0.687
	End	57.80	32.182	52.60	13.390	0.747
Alanine transaminase (ALT) (units/L)	Start	46.80	11.351	42.13	12.426	0.404
	End	48.80	18.431	45.33	21.068	0.777
Prothrombin time (seconds)	Start	10.29	1.816	9.77	1.964	0.419
	End	11.63	1.598	10.90	1.969	0.433
International normalized ratio	Start	1.00	0.000	1.08	0.277	0.289
	End	1.00	0.000	1.00	0.000	1
Activated partial thromboplastin time (seconds)	Start	27.29	5.525	27.08	5.795	0.918
	End	26.38	4.104	29.10	6.226	0.302
Serum sodium (mmol/L)	Start	138.81	4.969	139.77	3.632	0.591
	End	140.94	6.698	142.38	5.268	0.545
Serum potassium (mmol/L)	Start	3.88	0.500	3.85	0.555	0.879
	End	3.94	0.574	3.69	0.630	0.377
Serum chloride (mmol/L)	Start	110.50	7.294	106.77	6.126	0.157
	End	107.75	8.560	106.46	4.630	0.651
CO_2_ (mmol/L)	Start	20.36	4.396	19.36	7.953	0.693
	End	25.00	3.211	25.73	3.901	0.611
Blood glucose (mmol/L)	Start	6.67	1.676	8.58	4.100	0.18
	End	6.73	1.870	6.90	3.178	0.868
Blood urea nitrogen (mmol/L)	Start	2.50	1.211	3.54	1.391	0.048
	End	2.63	1.962	1.69	0.751	0.12
Serum creatinine (*µ*mol/L)	Start	32.25	11.947	37.15	7.069	0.208
	End	30.00	14.367	36.38	10.524	0.193

**Table 3:** Primary outcome in RCT. Comparison of dose(s) of fentanyl in groups. Groups compared via Student's *t*-test for comparison of means. *p* < 0.05 is significant. Values are expressed as mean and standard deviation.

Dose(s)	Group 1: dextromethorphan *µ* gram/kg/96 hrs (number: 16)	Group 2: placebo *µ* gram/kg/96 hrs (number: 13)	*p* value
Cumulative			
Mean	217.94	268	0.127
Std Dev	87.7	81.84	
SEM	21.9	22.7	
Infusion			
Mean	215.63	256.69	0.257
Std Dev	89.27	101.6	
SEM	22.32	28.2	
PRN			
Mean	2.31	3.62	0.101
Std Dev	2.21	1.85	
SEM	0.55	0.51	

## Abbreviations

DM: Dextromethorphan
PICU: Pediatric intensive care unit
NMDA: N-Methyl-D-aspartate.

## References

[1] Ricardo B. M., Adlaka M. R., Sehgal M. N. (2008). Opioid complications and side effects. *Pain Physician*.

[2] Erstad B. L., Puntillo K., Gilbert H. C. (2009). Pain management principles in the critically ill. *Chest*.

[3] Elliott K., Hynansky A., Inturrisi C. E. (1994). Dextromethorphan attenuates and reverses analgesic tolerance to morphine. *Pain*.

[4] Mao J., Price D. D., Caruso F. S., Mayer D. J. (1996). Oral administration of dextromethorphan prevents the development of morphine tolerance and dependence in rats. *Pain*.

[5] Mahmoodzadeh H., Movafegh A., Beigi N. M. (2010). Preoperative oral dextromethorphan does not reduce pain or morphine consumption after open cholecystectomy. *Middle East Journal of Anesthesiology*.

[6] Ilkjaer S., Bach L. F., Nielsen P. A., Wernberg M., Dahl J. B. (2000). Effect of preoperative oral dextromethorphan on immediate and late postoperative pain and hyperalgesia after total abdominal hysterectomy. *Pain*.

[7] Chau-In W., Sukmuan B., Ngamsangsirisapt K., Jirarareungsak W. (2007). Efficacy of pre- and postoperative oral dextromethorphan for reduction of intra- and 24-hour postoperative morphine consumption for transabdominal hysterectomy. *Pain Medicine*.

[8] King M. R., Ladha K. S., Gelineau A. M., Anderson T. A. (2016). Perioperative dextromethorphan as an adjunct for postoperative pain: a meta-analysis of randomized controlled trials. *Anesthesiology*.

[9] Savage S., Covington E. C., Heit H. A. (2001). *Definitions Related to the Use of Opioids for the Treatment of Pain*.

[10] Manworren R. C. B., Hynan L. S. (2003). Clinical validation of FLACC: preverbal patient pain scale. *Pediatric Nursing*.

[11] Tomlinson D., Von Baeyer C. L., Stinson J. N., Sung L. (2010). A systematic review of faces scales for the self-report of pain intensity in children. *Pediatrics*.

[12] Nilsson S., Finnström B., Kokinsky E. (2008). The FLACC behavioral scale for procedural pain assessment in children aged 5–16 years. *Paediatric Anaesthesia*.

[13] Sindrup S. H., Otto M., Finnerup N. B., Jensen T. S. (2005). Antidepressants in the treatment of neuropathic pain. *Basic and Clinical Pharmacology and Toxicology*.

[14] Boomershine C. S. http://www.painmedicinenews.com.

[15] Goldstein F. J. (2002). Adjuncts to opioid therapy. *The Journal of the American Osteopathic Association*.

[16] Wiffen P. J., Collins S., McQuay H., Carroll D., Jadad A., Moore A. (2005). Anticonvulsant drugs for acute and chronic pain. *Cochrane Database of Systematic Reviews*.

[17] Saarto T., Wiffen P. J. (2007). Antidepressants for neuropathic pain. *Cochrane Database of Systematic Reviews*.

[18] Dworkin R. H., O'Connor A. B., Backonja M. (2007). Pharmacologic management of neuropathic pain: evidence-based recommendations. *Pain*.

[19] Memis D., Inal M. T., Kavalci G., Sezer A., Sut N. (2010). Intravenous paracetamol reduced the use of opioids, extubation time, and opioid-related adverse effects after major surgery in intensive care unit. *Journal of Critical Care*.

[20] Singla N., Rock A., Pavliv L. (2010). A Multi-center, randomized, double-blind placebo-controlled trial of intravenous-ibuprofen (IV-ibuprofen) for treatment of pain in post-operative orthopedic adult patientspme. *Pain Medicine (United States)*.

[21] Micromedix Reference: Dextromethorphan, http://micromedex.com

[22] Hollander D., Pradas J., Kaplan R., McLeod H. L., Evans W. E., Munsat T. L. (1994). High-dose dextromethorphan in amyotrophic lateral sclerosis: phase I safety and pharmacokinetic studies. *Annals of Neurology*.

[23] Fleming P. M. (1986). Dependence on dextromethorphan hydrobromide. *British Medical Journal*.

[24] Capon D. A., Bochner F., Kerry N., Mikus G., Danz C., Somogyi A. A. (1996). The influence of CYP2D6 polymorphism and quinidine on the disposition and antitussive effect of dextromethorphan in humans. *Clinical Pharmacology and Therapeutics*.

[25] Price D. D., Mao J., Frenk H., Mayer D. J. (1994). The N-methyl-D-aspartate receptor antagonist dextromethorphan selectively reduces temporal summation of second pain in man. *Pain*.

